# A layer-wise fusion network incorporating self-supervised learning for multimodal MR image synthesis

**DOI:** 10.3389/fgene.2022.937042

**Published:** 2022-08-09

**Authors:** Qian Zhou, Hua Zou

**Affiliations:** School of Computer Science, Wuhan University, Wuhan, China

**Keywords:** self-supervised learning, medical image synthesis, feature-level fusion, generative adversarial network, encoder–decoder

## Abstract

Magnetic resonance (MR) imaging plays an important role in medical diagnosis and treatment; different modalities of MR images can provide rich and complementary information to improve the accuracy of diagnosis. However, due to the limitations of scanning time and medical conditions, certain modalities of MR may be unavailable or of low quality in clinical practice. In this study, we propose a new multimodal MR image synthesis network to generate missing MR images. The proposed model comprises three stages: feature extraction, feature fusion, and image generation. During feature extraction, 2D and 3D self-supervised pretext tasks are introduced to pre-train the backbone for better representations of each modality. Then, a channel attention mechanism is used when fusing features so that the network can adaptively weigh different fusion operations to learn common representations of all modalities. Finally, a generative adversarial network is considered as the basic framework to generate images, in which a feature-level edge information loss is combined with the pixel-wise loss to ensure consistency between the synthesized and real images in terms of anatomical characteristics. 2D and 3D self-supervised pre-training can have better performance on feature extraction to retain more details in the synthetic images. Moreover, the proposed multimodal attention feature fusion block (MAFFB) in the well-designed layer-wise fusion strategy can model both common and unique information in all modalities, consistent with the clinical analysis. We also perform an interpretability analysis to confirm the rationality and effectiveness of our method. The experimental results demonstrate that our method can be applied in both single-modal and multimodal synthesis with high robustness and outperforms other state-of-the-art approaches objectively and subjectively.

## 1 Introduction

Magnetic resonance imaging (MRI) has multiple modalities, such as T1 weighted (T1), T1 with contrast enhanced (T1-C), T2 weighted (T2), and T2-fluid-attenuated inversion recovery (T2-FLAIR) (Azad et al., 2022). Every modality shows specific pathological and structural information of the same organs. For example, T1 is usually used to explore the anatomical structure, while T2 is sensitive to bleeding and focuses more on lesions. In clinical diagnosis, doctors expect to comprehensively use the complementary information of different modalities to make more accurate and quicker decisions. However, because of the limitations of scanning costs, medical conditions, scanning time, and some other factors, it is difficult to obtain all multimodal MR images. In such a situation, doctors can only make a rough diagnosis of diseases, which may affect medical treatment. To solve this problem, many researchers are focusing on cross-modal medical image synthesis, which can synthesize images of the missing modality based on existing modal images. With this technology, patients do not need to conduct some expensive or damaged scans while doctors can still get corresponding medical images of patients, which may save much time and cost.

Presently, a large amount of work is based on the generative adversarial network (GAN) for medical image synthesis. Since the GAN (Goodfellow et al., 2014) was proposed in 2014, it has gained significant attention in image synthesis, including medical image synthesis. For example, the deep non-linear embedding deformation network (NEDNet) was proposed by Lin et al. (2022) for cross-modal brain MRI synthesis. Luo et al. (2022) presented an adaptive rectification based the GAN (AR-GAN) with a spectrum constraint to acquire high-quality standard-dose PET (SPET) images using low-dose PET (LPET) images. However, most of these studies focus on image synthesis from one modal to another. Multimodal image synthesis usually performs better than single modal image synthesis, since multimodal data contain more complementary information. Now some researchers have studied how to synthesize medical images from multi-source modalities. For example, Zhou et al. (2020) proposed a hybrid-fusion network (Hi-Net) for multimodal MR image synthesis. Alseelawi et al. (2022) proposed an effective strategy for multimodal medical image fusion based on a hybrid approach of a non-subsampled contourlet transform (NSCT) and a dual-tree complex wavelet transform (DTCWT).

However, several challenges remain for medical image synthesis. Unlike natural image synthesis, medical images are mostly 3D data, requiring massive computational resources in training. To reduce GPU memory usage, it is feasible to slice 3D data into 2D patches for training. For example, Osman and Tamam, (2022) trained a U-net model with 2D-paired MR images to perform image-to-image translation across MRI contrasts for the brain. Although this reduces the amount of computation and the demand for GPU memory, it also ignores part of the 3D contextual information. Jiao et al. (2020) proved that the combination of 3D information and 2D slices by self-supervised learning can effectively improve the quality of generated images, particularly image details. Another challenge for multimodal synthesis is how to effectively fuse the data from multiple sources. Both modality-specific characteristics and the common information of all modalities should be reserved through an effective fusion strategy. Although many fusion strategies have been designed to alleviate ineffective fusion to some extent, the common and unique information of different modalities are not well explored and modeled explicitly.

To address the aforementioned challenges, we propose a novel multimodal MR image synthesis network to generate missing MR images based on existing ones. Specifically, we pre-train the symmetric U-net (Ronneberger et al., 2015) backbone with 3D and 2D self-supervised learning tasks to take advantage of spatial contextual information. The backbone is based on auto-encoders to learn the most typical features of the sample in an encoding that uses the specified information capacity (Gao et al., 2022a). Then, the channel attention mechanism is involved in layer-wise fusion blocks to adaptively learn the best weights of multiple fusion operations. The fusion blocks learn the common representations of all modalities in shared latent space, while the features from the symmetric U-net (Ronneberger et al., 2015) present the modality-specific properties. In addition, we propose a GAN loss at both the feature-level and pixel-level to guarantee consistency between target images and generated images.

The main points of this study are summarized as follows:

• We use self-supervised learning tasks to take advantage of 3D and 2D auxiliary information during the feature extraction stage, in which the unique features of input modalities are better learned.
• We introduce a well-designed layer-wise fusion strategy to explore the correlations and obtain common features among various modalities effectively.
• We propose a novel GAN loss including the pixel-level loss to ensure that the generated images are realistic and clear subjectively, and the feature-level loss to ensure consistency between the generated images and real images in the anatomical characteristics.
• Comprehensive experimental evaluation shows that our model can generate high-quality MR images and perform better against other multimodal and single modal synthesis methods. The interpretability analysis verifies the correctness of our fusion model.

The rest of this study is organized as follows. We review some related works in Sec. 2. Sec. 3 describes the details of our approach for multimodal MR image synthesis. Then, we present several experiments to evaluate the superiority and interpretability of the proposed method in Sec. 4. Finally, we conclude the study in Sec. 5.

## 2 Related work

### 2.1 Medical image synthesis

Medical image synthesis is a popular topic in medical research, which aims to generate one imaging modality from other modalities. Classical methods are based on atlases or intensity transformation. Atlas-based methods perform deformation on the target modal atlas to synthesize target images, in which the deformation field is acquired by registering a source modal atlas to the source modal images (Lauritzen et al., 2019; Martinez-Girones et al., 2021). Martinez-Girones et al. (2021) proposed an approach to synthesize extended head and neck pseudo-CTs using an atlas comprising diverse anatomical overlapping MR-CT scans and deep learning methods. Intensity-based methods use intensity transformation to obtain the target images. A typical example of intensity-based methods is using image synthesis as an approach to solve sparse dictionary reconstruction, which is called dictionary learning. Huang et al. (2019) introduced the cross-modality dictionary learning scheme and a patch-based globally redundant model based on sparse representations to simultaneous super-resolution and cross-modality synthesis in brain MRI. However, the atlas-based method is sensitive to alignment accuracy and segmentation precision, thus requiring time-consuming manual labeling to obtain more accurate results. In the intensity-based methods, image patches at different scales are processed independently to reconstruct the dictionary. In addition, the predictions of patches are averaged during synthesis. Both factors may lead to the loss of high spatial frequency information and sub-optimal synthesis performance.

More recently, deep learning methods have achieved significant progress in medical image synthesis, particularly GAN-based approaches. The original GAN has inherent defects such as model collapse and gradient explosion, which have been addressed in the conditional GAN (CGAN) (Mirza and Osindero, 2014) and the deep convolutional GAN (DCGAN) (Radford et al., 2016). Pix2Pix (Isola et al., 2017) performs image-to-image translation pixel-to-pixel with paired data. CycleGAN (Zhu et al., 2017) extends Pix2Pix (Isola et al., 2017) to unpaired data with a cycle consistency loss. Fetty et al. (2020) took the StyleGAN (Karras et al., 2019) model as the generator for high-resolution medical image synthesis. However, these methods can only transform images from one domain to another but cannot use complementary information of multiple modalities for more accurate synthesis. Even though some methods (Liu et al., 2020; Bian et al., 2022) start to focus on multimodal image synthesis, they are not able to leverage 3D contextual information.

### 2.2 Self-supervised learning

Unlike supervised (Zhou et al., 2020) or weakly supervised learning (Xiao et al., 2021), self-supervised learning (Cao et al., 2020) usually learns the representations of the unlabeled data through a pretext task. It usually follows these steps: first, a pretext task is defined and the network is trained to solve this task to learn the representations. After that, the pre-trained model is fine-tuned for downstream tasks. Finally, the performance of these downstream tasks can be used to evaluate the quality of the features obtained through self-supervised learning.

According to different pretext tasks, self-supervised learning can be classified into three categories: context-based methods, contrastive learning-based methods, and generative model-based methods. Context-based methods aim to exploit the contextual information of the data, such as the order of words in natural language processing, the spatial structure information in image processing, the temporal information in video processing, etc. For example, Li et al. (2021) proposed JigsawGAN to learn semantic information and edge information of images, which is a GAN-based self-supervised method for solving jigsaw puzzles with unpaired images. Contrast learning (Tian et al., 2020; Wang et al., 2021; Dave et al., 2022) can be regarded as a discriminative method which aims to group positive samples and separate negative samples. Dave et al. (2022) developed a new temporal contrastive learning framework comprising local–local and local–global temporal contrastive loss to encourage the features to be distinct across the temporal dimension. Generative model-based approaches usually use some generative tasks as pretext tasks to learn features, such as image reconstruction (Fan et al., 2022), image inpainting (Quan et al., 2022), image coloring (Bi et al., 2021), etc. In this work, we use image inpainting and slice index prediction as pretext tasks to learn better representations of input modalities as detailed in Sec. 3.1.2.

### 2.3 Multimodal fusion

In the process of multimodal MR image synthesis, the information from different modalities needs to be fused. The commonly used fusion strategies can be divided into input-level fusion, feature-level fusion, and decision-level fusion (Zhou et al., 2019a). Input-level fusion is the channel-by-channel fusion treating multimodal images as multi-channel inputs. Ibtehaz and Rahman, (2020) proposed MultiResUNet for multimodal biomedical image segmentation, in which four MRI modalities are used as four different channels to obtain segmentation of brain tumors. Zhou et al. (2019b) embedded dilated convolution into 3D U-net for brain tumor segmentation in multi-parametric MRI, in which the multimodal images are stacked as different channels. Feature-level fusion (Zhan et al., 2021; Zuo et al., 2021; Gao et al., 2022b; Roy et al., 2022) extracts modality-specific features of each modality and then fuses them to use the complementary information. Zuo et al. (2021) proposed a deep auto-encoder multi-cascade fusion (DMC-Fusion) framework with classifier-based feature synthesis for automatically fusing medical multi-modalities. Zhan et al. (2021) developed multiple down-sampling branches corresponding to input modalities to specifically extract their unique features, and then fused them through a gate mergence mechanism to synthesize target images of MRI. Decision-level fusion fuses the results of each modality-specific network. For instance, Fu et al. (2021) introduced a multimodal spatial attention module to fuse the attention map from PET and the segmentation of CT to segment tumors.

Input-level fusion is the most commonly used and simplest fusion strategy for multimodal medical image synthesis, but it is difficult to take full advantage of the correlations and complementarity among multimodal images. Decision-level fusion is mostly used for tasks related to classification such as image segmentation and image recognition, which can be achieved by averaging the classification results or the majority voting strategy. Feature-level fusion is usually based on the assumption that different modal features share the same feature space. It is a big challenge to construct a shared latent space and build a fusion model based on feature correlations and modality complementarity (Zhang et al., 2021). Compared with input-level fusion and decision-level fusion, feature-level fusion can more effectively explore the relationship between different modal features.

In this work, all modalities are related to the same organ (i.e., the brain), and in the latent space, they may share some common features referring to the feature correlations. Furthermore, multimodal MR images show modality-specific properties due to different imaging contrasts, which are complementary to each other. Based on feature correlations and modality complementarity, an effective layer-wise fusion strategy is proposed to fuse features as described in Sec. 3.2.

## 3 Methods

In this section, we elaborate on three main components of the proposed approach in detail, that is, a feature extraction network for each modality, feature fusion network, and GAN-based generation network. The feature extraction network is used to extract the unique features of each modality; the feature fusion network takes the unique features of each modality as input and fuses them in the latent space to obtain the common features; and the generation network uses the unique and common features to synthesize a predicted image of target modality.

### 3.1 Feature extraction network

**FIGURE 1:**
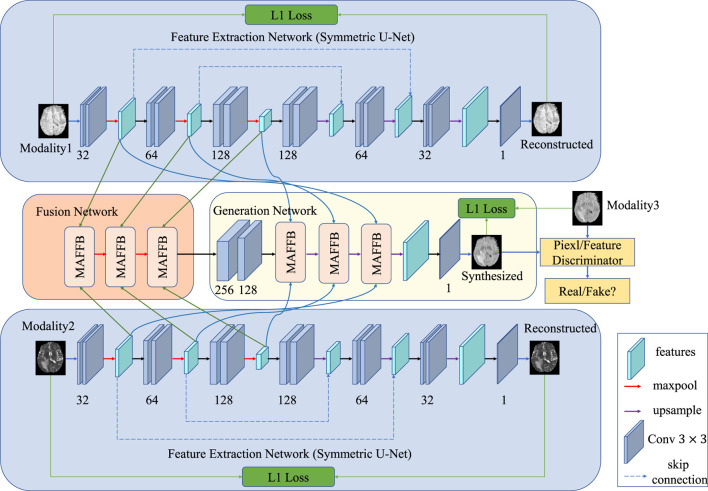
Pipeline of our proposed method. Our model comprises three main parts: the feature extraction network (a symmetric U-net pre-trained with self-supervised learning), the feature fusion network, and the GAN-based generation network. The feature extraction network learns the unique information of different modalities, while the fusion network aims to learn the common properties of multimodal images. The GAN-based generation network includes a generator and two discriminators. One discriminator distinguishes from the pixel-wise aspect, and the other discriminator considers the feature-level.

In multimodal medical image synthesis, there are both common and unique information among different modalities. As shown in Figure 1, to explore the correlations, we design a feature extraction network to learn the representations of input modalities. For each modality, the feature extraction network shares the same architecture with different parameter weights. As a result, the features of each modal should be in the same latent space, making them easier to fuse than raw data in different spaces. Specifically, the network is a symmetric auto-encoder with skip connections to reconstruct the source images. The deep features from the decoder and shallow features from the encoder are concatenated via skip connections to retain more detailed information.

#### 3.1.1 Architecture of feature extraction network

The feature extraction network aims to learn unique representations for each input modality. We develop a shared architecture and take the reconstruction task as the side-output supervision. The shared network architecture guarantees that the unique features of each modality have the same size and dimension in the shared latent space, which benefits the fusion stage. For the i-th modality, the input image is denoted as *x*_*i*_, and the extracted features from the encoder are defined as *f*_*i*_ = *EC*_*i*_(*x*_*i*_), where *EC*_*i*_ is the encoder. After that, the decoder reconstructs the original images from these features. To constrain the output, we adopt a pixel-wise l1-loss as the reconstruction loss function:

$$L^{\mathrm{Rec}}=\underset{i}{\sum }\|x_{i}-{\hat{x}}_{i}{\|}_{1},$$ (1)

where i denotes the i-th modality, ${\hat{x}}_{i}=DC_{i}\left(f_{i}\right)$ denotes the corresponding reconstructed image of *x*_*i*_, and *DC*_*i*_ is the decoder.

**FIGURE 2:**
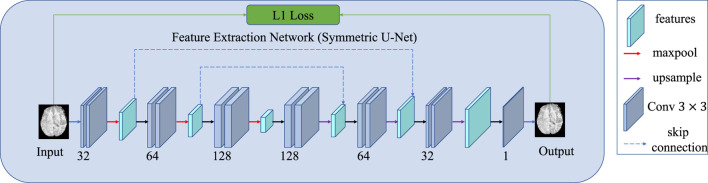
Detailed architecture of the feature extraction network, which can be considered as an encoder–decoder with skip connections.

A detailed schematic of the auto-encoder network is shown in Figure 2. The network is a symmetric U-Net with two skip connections between the pooling and upsampling layers. After each convolutional layer, a batch normalization layer is conducted in which the data are normalized using the mean and standard deviation computed from each batch. The activation functions of the encoder layers and the decoder layers are LeaklyRelu and Relu, respectively.

#### 3.1.2 Self-supervised pre-training

As mentioned previously, we take two self-supervised learning tasks to pre-train the network so that more 2D and 3D information can be used.

**FIGURE 3:**
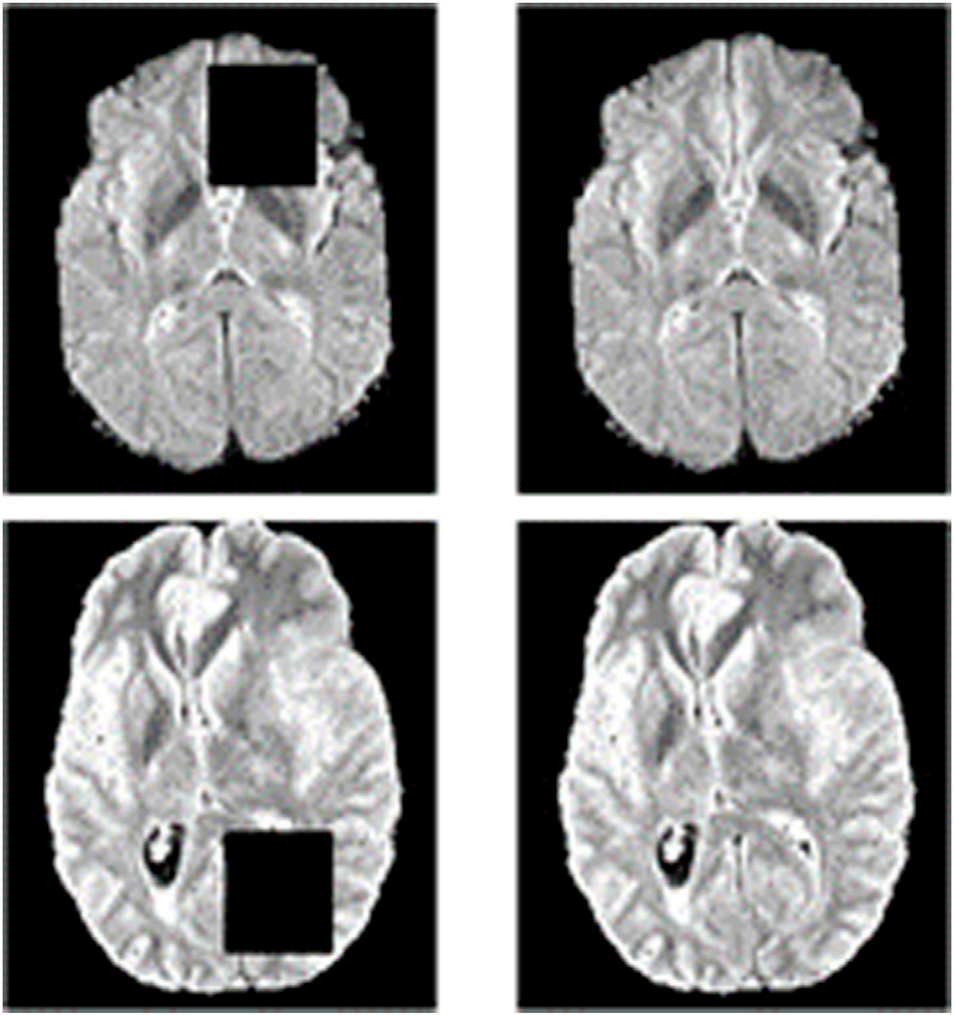
Schematic diagram of the image inpainting task. The left side is the result of masking part of the original image, and the right side is the original image. The image inpainting task aims to reconstruct the original image on the right through the input on the left.

Specifically, we take image inpainting as a pretext task to make full use of 2D contextual information. Some areas of the input image are covered by a mask in image inpainting. Then the network is trained to learn the contextual information and restore the image. A schematic diagram of the image inpainting task is shown in Figure 3. The only difference between reconstruction and inpainting is that some regions of the input are masked for image inpainting. Thus, the network structure and loss function for the image inpainting task are the same as the image reconstruction task.

**FIGURE 4:**
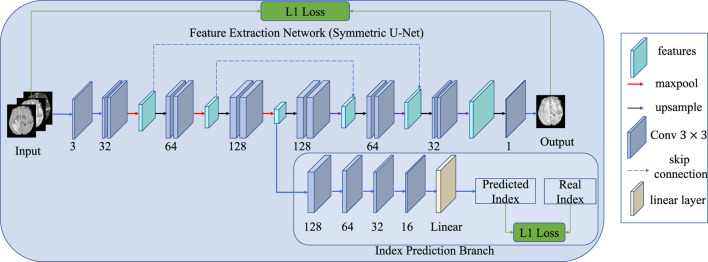
Schematic diagram of the index prediction task. Compared with the original network, an extra branch is used to predict the index of the present slice, and the input images contain three neighboring slices rather than one.

Similar to adjacent frames in video information, adjacent cross-sections of 3D medical data show correlations and continuity. Some methods use 2D slices to train the network to reduce the computation and the demand for GPU memory, but this cannot leverage the 3D information in the slicing direction. Therefore, we take the slice index prediction task as another self-supervised learning pre-training task, as shown in Figure 4. We assume that if the model can infer the position from adjacent slices, it means that the model has learned part of the whole anatomical structure and alleviated synthesis ambiguity. In such a situation, the input is not a single slice but three adjacent slices, in which three is a trade-off between the contextual information and the complexity of the network. Therefore, an additional convolutional layer is conducted before the feature extraction network. The additional convolutional layer compresses the input three slices into a single channel feature map without changing the size of the image. To predict the slice index, an index prediction branch is added to the decoder. The index prediction branch contains four convolution layers with batch normalization and ReLU activation, an average pooling layer, and a fully connected layer. The index prediction task can be regarded as a regression task. Finally, an index prediction loss is introduced in the reconstruction loss:

$$L^{\mathrm{Rec}}=\underset{i}{\sum }\left(\|x_{i}-{\hat{x}}_{i}{\|}_{1}+\|y_{i}-{\hat{y}}_{i}{\|}_{2}^{2}\right),$$ (2)

where *y*_*i*_ represents the real slice index of the i-th modal, and ${\hat{y}}_{i}$ represents the predicted slice index of the i-th modal.

### 3.2 Feature fusion network

After obtaining the representations of each modality in the same latent space, we can fuse the representations to explore the correlations among different modalities. To achieve this, in the feature fusion network, we introduced a layer-wise fusion block to learn the common features of all modalities. The input-level fusion strategy concatenates images of different modalities by channel and then feeds the fused result into a single network to get the final output. Unlike the input-level fusion strategy, the layer-wise fusion strategy is more complex to implement but can achieve better results. Specifically, both shallow and deep features from multiple layers can be fused explicitly in the layer-wise fusion strategy whereas only raw data can be fused implicitly in the input-level fusion strategy. Another advantage of the layer-wise fusion strategy is that features from our extraction networks share the same latent space with no gap. In contrast, raw data of each modality are in individual spaces with great diversities and gaps. Therefore a common representation can be learned easier using the layer-wise fusion strategy. Inspired by the powerful ability of attention mechanisms (Hu et al., 2018; Woo et al., 2018; Gao et al., 2021), we propose a multimodal attention feature fusion block (MAFFB) module to use the complementarity of different modalities. As can be seen in Figure 1, there are three MAFFB modules in the fusion network. Except for the first MAFFB module, each MAFFB module has three inputs including two unique representations from the encoder network and the output of the former MAFFB module. The MAFFB module can explore the correlations between both low-level and high-level features to learn common representations of all modalities.

**FIGURE 5:**
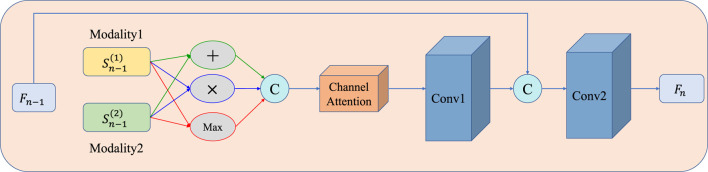
Structure of the MAFFB. Three common fusion operations (i.e., element-wise summation, element-wise product, and element-wise maximization) are included. *F*_*n*−1_ is the common features from the previous layer *n* −1. $S_{n-1}^{\left(1\right)}$ and $S_{n-1}^{\left(2\right)}$ are the unique features of two input modalities from layer *n*−1. C means concatenation. *F*_*n*_ is the obtained common features of the present layer *n*.

A detailed illustration of the MAFFB module is shown in Figure 5, in which channel attention guidance is applied to adaptively weigh three popular fusion operations (i.e., element-wise summation, element-wise product, and element-wise maximization). There is no evidence to show which of the three is better, therefore, we use all of them. $S_{n-1}^{\left(i\right)}\in R^{C\times H\times W}$ denotes the features from the (n-1)-th layer of the i-th modality (i = 1, 2), where C is the number of feature channels, and W and H are the width and height of the feature maps, respectively. The results of the three operations are

$$F_{+}=S_{n-1}^{\left(1\right)}+S_{n-1}^{\left(2\right)},$$ (3)

$$F_{\times }=S_{n-1}^{\left(1\right)}\times S_{n-1}^{\left(2\right)},$$ (4)

$$F_{\max}=Max\left(S_{n-1}^{\left(1\right)},S_{n-1}^{\left(2\right)}\right),$$ (5)

where “+ ,” “×,” and “*Max*” denote element-wise summation, element-wise product, and element-wise maximization operations, respectively. Following that, the results are stacked as different channels:

$$F_{\mathrm{concat}}=\left[F_{+},F_{\times },F_{\max}\right]\in R^{3C\times H\times W}.$$ (6)

**FIGURE 6:**
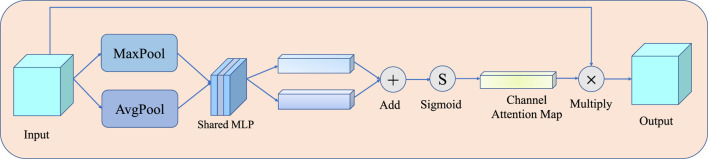
Illustration of the channel attention module, which is used to adaptively weigh the results of three fusion operations. The shared MLP comprises 1×1 Conv, Relu, and 1×1 Conv.

Then, *F*_*concat*_ is fed into a channel attention module (detailed in Figure 6) to obtain the channel attention map. The channel attention module can be divided into two steps: first, average pooling and max pooling are conducted as Eq. 7– Eq. 8; then, the results are fed into a shared multilayer perceptron to compute the attention map as Eq. 9.

$$AVGPool\left(x_{k}\right)=\frac{1}{H\times W}\overset{H}{\underset{i=1}{\sum }}\overset{W}{\underset{j=1}{\sum }}x_{k}\left(i,j\right),$$ (7)

$$MAXPool\left(x_{k}\right)=Max\left(x_{k}\left(i,j\right)\right),\;i=1,2,\ldots ,H;j=1,2,\ldots ,W,$$ (8)

$$M_{c}\left(F\right)=\sigma \left(f^{1\times 1}\left(\delta \left(f^{1\times 1}\left(AVGPool\left(x_{k}\right)\right)\right)\right)+f^{1\times 1}\left(\delta \left(f^{1\times 1}\left(MAXPool\left(x_{k}\right)\right)\right)\right)\right),$$ (9)

where *x*_*k*_ (*i*, *j*) represents the value of the *k*th channel in *F*_*concat*_ at position (i,j). *AVGPool* (*x*_*k*_) and *MAXPool* (*x*_*k*_) represent the global average pooling and global maximum pooling on the k-th channel of *F*_*concat*_, respectively. *f* ^1 × 1^ represents a 1 × 1 convolution operation, *δ* is the ReLu activation function, and *σ* is the Sigmoid activation function. Then, the attention map is multiplied by *F*_*concat*_ and the results are fed into the first convolutional layer. After that, the output of the first convolutional layer is concatenated with output *F*_*n*−1_ of the previous MAFBB module and then fed into the second convolutional layer. Finally, the output *F*_*n*_ of the present MAFBB module is obtained, which is the common representation of the present layer. In each MAFFB, we introduce a batch normalization layer after each convolution layer with the Relu activation function.

### 3.3 GAN-based generation network

Once the modality-specific features and common representations of all modalities are obtained, we can use them to synthesize the image of the target modality. We design a GAN-based generation network comprising two parts: one generator and two discriminators. The generator G can be roughly regarded as a decoder model and tries to generate an image to confuse the discriminator D while the discriminator D identifies the generated image from the real image.

For generator G, the modality-specific features fused by the feature fusion network are compressed into channels by two layers of convolution to reduce the computation. Then three MAFFBs are used to further fuse the features. To retain more detailed information in the generated image, the input of the MAFFB contains the common representations fused by the feature fusion network and the unique features extracted by the feature extraction network. The output of the last MAFFB is passed through an upsampling and a convolutional layer to obtain the synthetic image. Each convolution layer is followed by a batch normalization layer and a Relu activation function.

The proposed synthetic network contains two discriminator networks: one considers pixel-level loss and the other one is trained with feature-level loss. For the discriminator with pixel-level loss, the network architecture is shown in Table 1. We take a 2D image of size 128 × 128 as input. The network contains five convolutional layers, each of which is followed by a batch normalization layer and a LeaklyReLu activation function with a slope of 0.2. For the discriminator with feature-level, the network also contains five convolutional layers as shown in Table 2. Compared with the pixel-level discriminator, its input is the edge information extracted from CannyNet^1^. CannyNet is used to extract anatomical features, which implements the Canny (Canny, 1986) edge detection algorithm. The pixel-level discriminator ensures that generated images are more realistic in appearance, while the feature-level discriminator constrains that generated images and real images are anatomically consistent.

**TABLE 1:** Architecture of the pixel-level discriminator.

Layer	Input	Parameters	Activation
dis_1	images	Conv (3 × 3,32), BN, stride = 2	LeakyRelu, 0.2
dis_2	dis_1	Conv (3 × 3,64), BN, stride = 2	LeakyRelu, 0.2
dis_3	dis_2	Conv (3 × 3,128), BN, stride = 2	LeakyRelu, 0.2
dis_4	dis_3	Conv (3 × 3,256), BN, stride = 2	LeakyRelu, 0.2
output	dis_4	Conv (3 × 3,1), BN, stride = 1	LeakyRelu, 0.2

**TABLE 2:** Architecture of the feature-level discriminator.

Layer	Input	Parameters	Activation
dis_1	features	Conv (3 × 3,32), BN, stride = 2	Relu, 0.2
dis_2	dis_1	Conv (3 × 3,64), BN, stride = 2	Relu, 0.2
dis_3	dis_2	Conv (3 × 3,128), BN, stride = 2	Relu, 0.2
dis_4	dis_3	Conv (3 × 3,256), BN, stride = 2	Relu, 0.2
output	dis_4	Conv (3 × 3,1), BN, stride = 1	Relu, 0.2

In generation networks, the loss function contains three components: pixel-level reconstruction loss between the real image and the generated image as Eq. 10, pixel-level generative adversarial loss as Eq. 11, and feature-level generative adversarial loss as Eq. 12.

$$L^{\mathrm{GRec}}=\|y-G\left(x_{1},x_{2}\right){\|}_{1},$$ (10)

$$L_{\mathrm{pixel}}^{\mathrm{adv}}=\underset{G}{\min}\underset{D}{\max}E_{y∼p_{\mathrm{data}}}\left[\log\left(\;\right.1-D_{\mathrm{pixel}}\left(y\right)\right]+E_{x_{1},x_{2}∼p_{\mathrm{data}}}\left[\log\left(\;\right.1-D_{\mathrm{pixel}}\left(\;\right.G\left(x_{1},x_{2}\right)\right],$$ (11)

$$L_{\mathrm{feature}}^{\mathrm{adv}}=\underset{G}{\min}\underset{D}{\max}E_{y∼p_{\mathrm{data}}}\left[\log\left(\;\right.1-D_{\mathrm{feature}}\left(C\left(y\right)\right)\right]+E_{x_{1},x_{2}∼p_{\mathrm{data}}}\left[\log\left(\;\right.1-D_{\mathrm{feature}}\left(\;\right.C\left(G\left(x_{1},x_{2}\right)\right)\right],$$ (12)

where *G* (*x*_1_, *x*_2_) is the generated image, y is the ground truth, *D*_*pixel*_ is the pixel-level discriminator, *D*_*feature*_ is the feature-level discriminator, and C is the CannyNet.

Finally, the total loss function of the whole network is

$$L=L_{\mathrm{pixel}}^{\mathrm{adv}}+L_{\mathrm{feature}}^{\mathrm{adv}}+\lambda_{1}L^{\mathrm{GRec}}+\lambda_{2}L^{\mathrm{Rec}},$$ (13)

where *λ*_1_ and *λ*_2_ are non-negative trade-off parameters.

## 4 Experiments

In this section, we first describe our dataset and evaluation metrics. Then, we present our results and compare them with other methods. Especially, we perform an interpretability analysis of the proposed model.

### 4.1 Datasets

We use the Brain Tumor Segmentation Challenge 2018 (BraTS 2018) dataset (Menze et al., 2014) for the training and evaluation of our method. The dataset contains MR brain image data of 285 cases, and comprises four MRI modalities: T1, T1c, T2, and T2-Flair. The size of each MR image is 240×240×155, and all corresponding multimodal data have been registered. We divide 80% of the 285 samples as the training set and the remaining 20% as the test set. We use 2D axial-plane slices for training. Because the boundary part of the 2D slice contains a lot of invalid information (i.e., the intensity of the boundary part is 0), only the central area of 160×180 is used. At the same time, to expand the dataset, each 160×180 area is cropped into four overlapping 128 × 128 image blocks. The overlapping part adopts the strategy of averaging in the final synthesis. In addition, the intensity of all training data is scaled to [-1, 1]. When performing self-supervised pre-training on the feature extraction network, we use four masks of size 32 × 32.

### 4.2 Evaluations

To evaluate the effectiveness of our method, we compare different variants of the proposed method with the Hi-Net (Zhou et al., 2020) model and MM-Syns (Chartsias et al., 2017), both of which are proposed for multimodal MR brain image synthesis. There are four variants of our proposed method: variant one contains only self-supervised learning pre-training, variant two contains only the attention mechanism, variant three contains only feature-level constraints, and the final variant four contains all the aforementioned three highlights at the same time. Moreover, our method is also compared with Pix2Pix (Isola et al., 2017), CycleGAN (Zhu et al., 2017), and StyleGAN (Karras et al., 2019) for single modal synthesis. For the quality evaluation, we use the peak signal-to-noise ratio (PSNR), the mean square error (MSE), and the structure similarity (SSIM) as the metrics. Both the PSNR and MSE are image quality evaluation indicators based on image pixel statistics. Supposing the ground truth is y(x) and the synthesized image is G(x), the quantity metrics are defined as follows:

$$MSE=\frac{1}{N}\|y\left(x\right)-G\left(x\right){\|}_{2}^{2},$$ (14)

$$PSNR=10log_{10}\frac{\max^{2}\left(y\left(x\right),G\left(x\right)\right)}{MSE},$$ (15)

where max^2^ (*y*(*x*), *G*(*x*)) represents the square of the maximum intensity in y(x) and G(x).

As for SSIM, it measures the degree of similarity between the ground truth and the synthesized image. SSIM is calculated as follows:

$$SSIM=\frac{\left(2\mu_{y\left(x\right)}\mu_{G\left(x\right)}+c_{1}\right)\left(2\sigma_{y\left(x\right)G\left(x\right)}+c_{2}\right)}{\left(\mu_{y\left(x\right)}^{2}+\mu_{G\left(x\right)}^{2}+c_{1}\right)\left(\sigma_{y\left(x\right)}^{2}+\sigma_{G\left(x\right)}^{2}+c_{2}\right)},$$ (16)

where $\mu_{y\left(x\right)},\mu_{G\left(x\right)},\sigma_{y\left(x\right)}^{2},\sigma_{G\left(x\right)}^{2}$ are the means and variances of y(x) and G(x), *σ*_*y*(*x*)*G*(*x*)_ is the covariance of images y(x) and G(x), and *c*_1_ and *c*_2_ are two positive constants to avoid dividing by 0. It is worth noting that lower MSE, higher PSNR, and higher SSIM are what we expected.

### 4.3 Results

We divide the experiments into two groups, one for multimodal synthesis, and the other for single modal synthesis.

**FIGURE 7:**
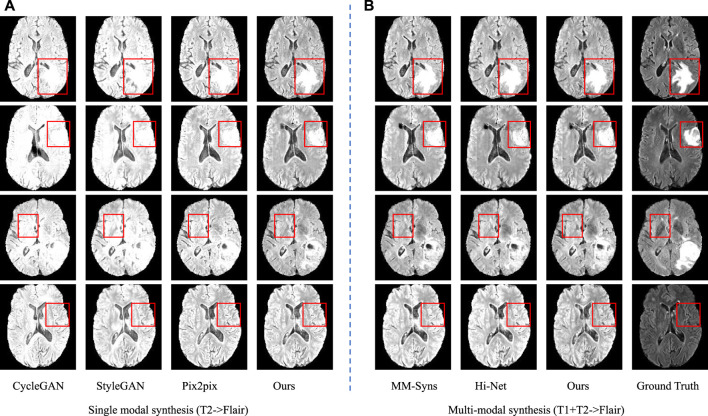
Synthesis results of single modal and multimodal. **(A)** is the sing modal results using T2 to synthesize Flair; **(B)** is the multimodal results using T1 and T2 to synthesize Flair.

In the first group, we take T1 and T2 as input to synthesize the T2-Flair modality and compare our method with Hi-Net (Zhou et al., 2020) and MM-Syns (Chartsias et al., 2017). Table 3 shows the quantitative evaluation results. As we can see, our method outperforms any other multimodal MR image synthesis approach. This suggests that our method can effectively explore the correlations and meanwhile preserve the modality-specific properties, which are essential to the synthesis performance. Figure 7B shows the subjective results of our method compared with others. As can be seen, more details and fewer blurred areas can be found in our method. Hi-net (Zhou et al., 2020) is better than MM-Syns (Chartsias et al., 2017) for its hybrid fusion strategies. Compared with Hi-Net (Zhou et al., 2020) and MM-Syns (Chartsias et al., 2017), our method can leverage more 2D and 3D contextual information and use a more effective fusion strategy to improve performance.

**TABLE 3:** Comparison results of the objective evaluation on multimodal synthesis (T1+T2→Flair).

Methods	MSE *↓*	PSNR *↑*	SSIM *↑*
MM-Syns Chartsias et al. (2017)	0.0379	24.12	0.8723
Hi-Net Zhou et al. (2020)	0.0244	25.67	0.9034
Ours	**0.0224**	**27.41**	**0.9272**

In the second group, T2 is used to synthesize T2-Flair to evaluate the performance of single modal synthesis. In this situation, the inputs of two feature extraction networks in our method are both T2 images. The compared models include Pix2Pix (Isola et al., 2017), CycleGAN (Zhu et al., 2017), and StyleGAN (Karras et al., 2019). Table 4 and Figure 7A show the objective and subjective comparison results for different methods. It can be seen that StyleGAN (Karras et al., 2019) and CycleGAN (Zhu et al., 2017) only synthesize some fuzzy areas, most of which have low contrast and extremely low retention of detailed information. Pix2Pix (Isola et al., 2017) is much better than CycleGAN (Zhu et al., 2017) and StyleGAN (Karras et al., 2019) for its strong supervision. In this group, multimodal data are not available; thus, we are not allowed to explore the complementarity of multiple modalities. Since 2D and 3D contextual information are better used in our method, we can still achieve the best results both quantitatively and qualitatively. Overall, using self-supervised learning tasks to make use of 2D and 3D auxiliary information can significantly improve performance.

**TABLE 4:** Comparison results of the objective evaluation on single modal synthesis (T2 → Flair).

Methods	MSE *↓*	PSNR *↑*	SSIM *↑*
Pix2Pix Isola et al. (2017)	0.0429	22.96	0.8631
CycleGAN Zhu et al. (2017)	0.0507	22.54	0.8322
StyleGAN Karras et al. (2019)	0.0473	22.77	0.8451
Ours	**0.0394**	**23.41**	**0.8772**

Compared with other state-of-the-art methods, the experimental results in multimodal synthesis demonstrate the superiority of our method. This is mainly because of our powerful feature extraction and effective feature fusion strategy. MM-Syns (Chartsias et al., 2017) uses an encoder to learn modality-specific features and max operation to fuse the features. The simple max operation may lose some detailed information, and thus is unable to effectively explore and use the correlations and complementarity between multimodal features. Hi-Net (Zhou et al., 2020) uses a more complex fusion strategy with three different common fusion operations, which can better explore complementary information from multiple modalities and exploit their correlations to improve synthesis performance. However, Hi-Net (Zhou et al., 2020) only applies 2D slices to synthesize the target modality, ignoring the 3D information in the medical image during the feature extraction process. Our method not only uses self-supervised learning tasks to pre-train the model to use 3D and 2D contextual information but also uses more complex fusion strategies to fuse features of different modalities. Self-supervised pre-training enables our model to learn more effective features from different modalities. The efficient fusion method can better exploit the correlations among features to fuse them in the latent feature space. As a result, the unique features and common features of multiple modalities are well preserved in the synthesis results. Moreover, the proposed method is a robust model, as it can be applied in both single modal and multimodal synthesis.

### 4.4 Ablation study

To verify the effectiveness of our three highlights, four variants of our method are provided for ablation studies. The experimental results are shown in Table 5. From the experimental results, it can be seen that the pre-training model with self-supervised learning tasks achieves better performance than the attention mechanism and feature-level loss, showing the importance of using 3D and 2D contextual information.

**TABLE 5:** Comparison results of the objective evaluation on different variants of our method (T1+T2 → Flair).

Methods	MSE *↓*	PSNR *↑*	SSIM *↑*
Variant 1 (self-supervised learning)	0.0231	26.97	0.9228
Variant 2 (channel attention)	0.0237	26.34	0.9177
Variant 3 (feature-level discriminator)	0.0249	25.92	0.9089
Variant 4 (all above)	**0.0224**	**27.41**	**0.9272**

We analyze the reasons for this result as follows:

1) Jiao et al. (2020) confirmed that 3D contextual information can effectively improve the performance of single modal medical image synthesis. In this study, we verify the importance of 3D contextual information in multimodal medical image synthesis through experimental results. Our model can take advantage of 3D and 2D contextual information through pre-training to significantly improve the quality of the synthesized image.
2) The attention mechanism improves the fusion strategy. When adopting a variety of fusion operations, we use the attention mechanism to adaptively adjust the fusion weights. As a result, the model can more efficiently use the correlations and complementarity to fuse features. Both the unique attributes of a single modality from the feature extraction network and common information of multiple modalities from the fusion network can be well retained during the synthesis to improve performance.
3) The feature-level loss constrains the extracted anatomical structure features, which requires CannyNet (Canny, 1986) to extract edge features, and an additional discriminator is used to distinguish the extracted features to ensure the consistency of anatomical structures.

### 4.5 Interpretability analysis

Experimental results have demonstrated the superiority and robustness of our method compared with other state-of-the-art methods for MR image synthesis. To verify the reasonability of our proposed fusion strategy, we visualize the unique features of each input modality (features from auto-encoders) and common features of all modalities (output of MAFFB).

**FIGURE 8:**
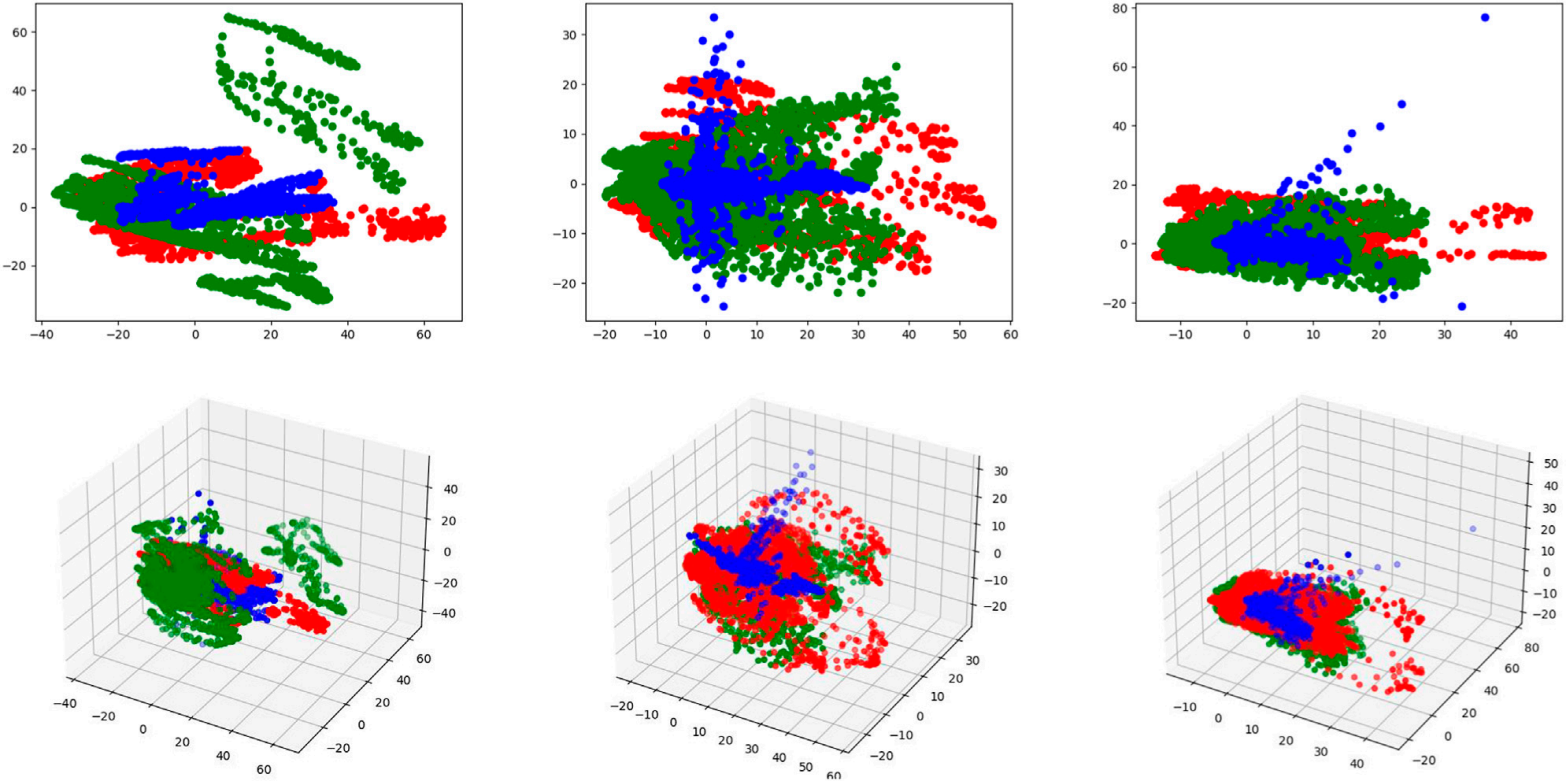
Visualizations of the unique and common features in 3D and 2D latent spaces. Red and green points represent the unique features of two input modalities, and blue points represent the common features. The first row represents the 2D latent space and the second row represents the 3D latent space. From left to right, each column represents *MAFFB*_1_, *MAFFB*_2_, and *MAFFB*_3_, respectively.

**FIGURE 9:**
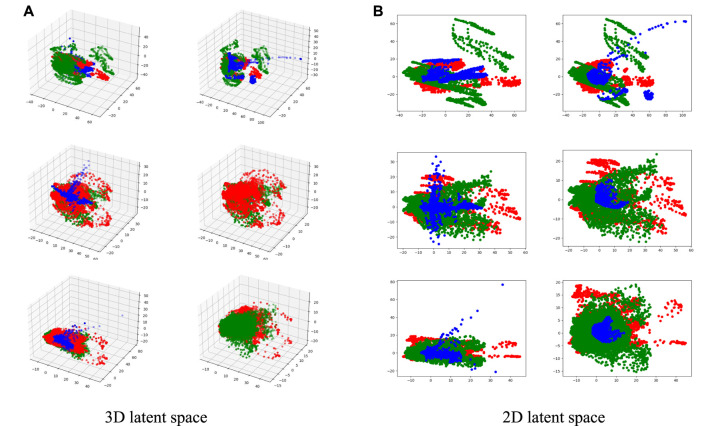
Visualizations of the unique and common features in 2D and 3D latent spaces. Red and green points represent the unique features of two input modalities, and blue points represent the common features. In **(A)** and **(B)**, the left column represents *MAFFB*_*i*_ in the fusion network, and the right column represents *MAFFB*_6−*i*_ in the GAN-based network, i = 1, 2, 3.

We have six MAFFB modules (denoted as *MAFFB*_1_, ..., *MAFFB*_6_) in our model. The first three modules (*MAFFB*_1_, *MAFFB*_2_, and *MAFFB*_3_) are in the fusion network, and the others (*MAFFB*_4_, *MAFFB*_5_, and *MAFFB*_6_) are in the generation network. As mentioned before, unique features are learned through the feature extraction network, and the fusion network takes the unique features as input to obtain the common features. We project the unique features (i.e., $S_{n-1}^{\left(1\right)}$ and $S_{n-1}^{\left(2\right)}$ in Figure 5) and common features (i.e., *F*_*n*_ in Figure 5) of all MAFFB modules into the 3D and 2D latent space by the principal component analysis in Figure 8 and Figure 9.

Figure 8 shows the distributions of the unique and common features of the three MAFFB modules in the feature fusion network. In Figure 8, it can be seen that the common features are located in the middle area of the unique features of the two input modalities. As the depth of the network increases, the common features get closer to the intersection of the unique features.

As shown in Figure 1, *MAFFB*_*i*_ in the fusion network and *MAFFB*_6−*i*_ (i = 1, 2, 3) in the generation network share the same unique features as input. Therefore, the common features of *MAFFB*_*i*_ and *MAFFB*_6−*i*_ are compared and the visualizations can be found in Figure 9. Obviously, the common features in the generative network are more concentrated among the unique features than those in the fusion network. These visualization results demonstrate that the MAFFB module can fuse unique features in an effective way to obtain common features, and as the depth of the network increases, the fusion improves. Because of the high performance of our proposed layer-wise fusion network, the correlations and complementarity of modalities can be well used in our model.

### 4.6 Discussion

Our method is effective and robust and can be applied in many ways. One potential application is to help doctors make more accurate clinical diagnoses. T1ce needs to inject contrast media into the patient during imaging. Using our proposed method, patients may not need to conduct such a damaged scan, yet doctors can still obtain the images of T1ce to make a more comprehensive diagnosis. In addition, our method can be applied for data augmentation. As is known to us, in deep learning, most approaches are data-driven. But in practice, it is a big challenge to obtain a large amount of training data, particularly when the technique to obtain data is damaged and newly invented. In this case, our method can be used to synthesize missing or imbalanced modal images.

However, there exist some shortcomings in our proposed method. First, our method requires self-supervised pre-training, which will increase the training time as shown in Table 6. Second, since the input data of our method are registered by experts, we do not explore our method on unpaired or not strictly aligned data. We hope future researchers can propose an end-to-end way to use 3D contextual information while reducing the computational cost of 3D medical data. In addition, weakly supervised learning may be a good choice since paired and registered data are difficult to obtain in practice.

**TABLE 6:** Training cost comparison between our method and Hi-Net Zhou et al. (2020) on four RTX 3090 GPUs, and the batch size of 3D volume is set to 1.

Methods	Memory (MB)	Time
2D and 3D self-supervised joint pre-training	25,890	15 h
Ours	67,400	41.3 h
Hi-Net Zhou et al. (2020)	65,852	40 h

## 5 Conclusion

In this study, we have proposed a new approach with the layer-wise fusion strategy to synthesize target modal MR images using multimodal MR images. The proposed method combines self-supervised learning with generative adversarial networks. Specifically, modality-specific features are first extracted from an auto-encoder, which is pre-trained with self-supervised learning tasks to better use 2D and 3D contextual information. Then a fusion network is used to explore the correlations across multiple modalities and fuse the features from different layers. Moreover, a channel attention mechanism is used in the layer-wise MAFFB to adaptively weigh three widely used fusion operations. Finally, a GAN-based network with two discriminators is introduced to synthesize the target image with both common and unique information of all modalities. Experimental results demonstrate the superiority of our method both qualitatively and quantitatively, in comparison to other state-of-the-art synthesis methods. In the future, we will explore whether this method can be applied in cross-modal synthesis with unpaired or not strictly aligned data.

## Data Availability

The original contributions presented in the study are included in the article/Supplementary Material; further inquiries can be directed to the corresponding author.

## Footnotes

1
https://github.com/DCurro/CannyEdgePytorch
